# Sensitive and Specific Detection of Low-Level Antibody Responses in Mild Middle East Respiratory Syndrome Coronavirus Infections

**DOI:** 10.3201/eid2510.190051

**Published:** 2019-10

**Authors:** Nisreen M.A. Okba, V. Stalin Raj, Ivy Widjaja, Corine H. GeurtsvanKessel, Erwin de Bruin, Felicity D. Chandler, Wan Beom Park, Nam-Joong Kim, Elmoubasher A.B.A. Farag, Mohammed Al-Hajri, Berend-Jan Bosch, Myoung-don Oh, Marion P.G. Koopmans, Chantal B.E.M. Reusken, Bart L. Haagmans

**Affiliations:** Erasmus Medical Center, Rotterdam, the Netherlands (N.M.A. Okba, V.S. Raj, C.H. GeurtsvanKessel, E. de Bruin, F.D. Chandler, M.P.G. Koopmans, C.B.E.M. Reusken, B.L. Haagmans);; Utrecht University, Utrecht, the Netherlands (I. Widjaja, B.-J. Bosch);; Seoul National University College of Medicine, Seoul, South Korea (W.B. Park, N.-J. Kim, M.-D. Oh);; Ministry of Public Health, Doha, Qatar (E.A.B.A. Farag, M. Al-Hajri)

**Keywords:** Middle East respiratory syndrome coronavirus, diagnostics, ELISA, spike, MERS, S1, human, camels, coronavirus, antibodies, neutralizing, serology, viruses, the Netherlands, South Korea, Qatar, MERS-CoV

## Abstract

Middle East respiratory syndrome coronavirus (MERS-CoV) infections in humans can cause asymptomatic to fatal lower respiratory lung disease. Despite posing a probable risk for virus transmission, asymptomatic to mild infections can go unnoticed; a lack of seroconversion among some PCR-confirmed cases has been reported. We found that a MERS-CoV spike S1 protein–based ELISA, routinely used in surveillance studies, showed low sensitivity in detecting infections among PCR-confirmed patients with mild clinical symptoms and cross-reactivity of human coronavirus OC43–positive serum samples. Using in-house S1 ELISA and protein microarray, we demonstrate that most PCR-confirmed MERS-CoV case-patients with mild infections seroconverted; nonetheless, some of these samples did not have detectable levels of virus-neutralizing antibodies. The use of a sensitive and specific serologic S1-based assay can be instrumental in the accurate estimation of MERS-CoV prevalence.

Middle East respiratory syndrome coronavirus (MERS-CoV) poses a public health threat; ongoing outbreaks have been reported since its detection in 2012 (*1*). MERS-CoV infection may be asymptomatic or may cause illness ranging from mild to fatal; fatal infections account for 35% of reported cases (*2*–*5*). Dromedary camels are the virus reservoir (*6*,*7*) and pose a high risk of infecting humans in contact with them (*4*,*7*–*9*). These spillover events may seed outbreaks in the community (*10*), which occur mainly in healthcare settings (*11*,*12*) and, to a lesser extent, among patient household contacts (*13*–*15*). Although not sustained, human-to-human transmission accounts for most reported cases (*16*) and may initiate outbreaks outside endemic areas, as seen in the 2015 South Korea outbreak (*17*). However, the rate of human-to-human transmission and total disease burden of MERS-CoV are not fully clear because we lack accurate data on the frequency of asymptomatic and mild infections.

Diagnostic assays with validated high sensitivity and specificity are crucial to estimate the prevalence of MERS-CoV. Molecular-based assays have been developed that enable sensitive and specific diagnosis of MERS-CoV infections (*18*,*19*). Although the molecular detection of viral nucleic acid by reverse transcription PCR (RT-PCR) is the standard for MERS-CoV diagnosis, serologic detection remains necessary. Viral nucleic acid is detectable within a limited timeframe after infection, and samples from the lower respiratory tract are required for reliable results. Furthermore, whereas mutations in the viral regions where the PCR probes bind could lead to decreased sensitivity (*20*), genetically diverse MERS-CoV strains may retain antigenic similarity (*21*). Validated serologic assays are needed to ensure that the full spectrum of infections is identified; antibodies can be detected for longer periods after infection and even if viruses mutate. Several research groups and companies have developed serologic assays allowing for high-throughput surveillance for MERS-CoV infections among large populations (*15*,*19*,*22*–*25*).

Despite the number of serological assays developed, none is considered to be fully validated. There are 2 major challenges concerning specificity and sensitivity aspects of MERS-CoV serologic assays. The first challenge is that 90% of the human population have antibodies against common cold–causing human coronaviruses (HCoVs) that could cross-react, resulting in false positives in serologic assays, especially in persons infected with viruses belonging to the same genus of β-coronaviruses as human seasonal coronaviruses OC43 and HKU1 (*26*). The spike protein, specifically its N-terminal S1 domain, is highly immunogenic and divergent among HCoVs, so it is an ideal candidate for virus-specific serologic assays (*27*). The second challenge is the low antibody responses among mildly infected and asymptomatic cases. Severe MERS-CoV infections result in a robust immune response allowing serologic detection in patients with positive or negative PCR outcomes (*28*), but PCR-diagnosed mild or asymptomatic infections may cause variable immune responses that can be undetectable by serologic assays (*5*,*15*,*17*). Therefore, a sensitive assay is necessary to avoid false-negative results that can cause failure in detection of subclinical infections and underestimation of prevalence in serosurveillance studies. We evaluated the antibody responses following severe and mild laboratory-confirmed MERS-CoV infections, validating and comparing different assay platforms for the specific and sensitive detection of MERS-CoV infections.

## Materials and Methods

### Serum Samples

We used a total of 292 serum samples in this study (Table 1; Appendix). The samples represented patients with serologically identified (*8*) and PCR-confirmed MERS-CoV infections (*17*,*29*), a cohort of healthy blood donors as a control group, and patients confirmed by RT-PCR to have non–MERS-CoV respiratory virus infections to assess assay specificity. The use of serum samples from the Netherlands was approved by the Erasmus Medical Center local medical ethics committee (MEC approval 2014-414). The Institutional Ethics Review Board of Seoul National University Hospital approved the use of samples from patients in South Korea (approval no. 1506–093–681). The Ethics and Institutional Animal Care and Use Committees of the Medical Research Center, Hamad Medical Corporation, approved the use of samples from Qatar (permit 2014–01–001).

**Table 1:** Cohorts used in study of specificity and sensitivity of assays for MERS-CoV*

Cohort	Country	Sample source	Infection	No. samples	Postdiagnosis range
A	The Netherlands	Healthy blood donors (negative cohort)	NA	50	NA
B	The Netherlands	Non-CoV respiratory infections†	Adenovirus	5	2–4 w
			Bocavirus	2	2–4 w
			Enterovirus	2	2–4 w
			HMPV	9	2–4 w
			Influenza A	13	2–4 w
			Influenza B	6	2–4 w
			Rhinovirus	9	2–4 w
			RSV	9	2–4 w
			PIV-1	4	2–4 w
			PIV-3	4	2–4 w
			*Mycoplasma pneumoniae*	1	2–4 w
			CMV	9	2–4 w
			EBV	12	2–4 w
C	The Netherlands	Persons with recent CoV infections†	α-CoV HCoV-229E	19	2 w–1 y
			α-CoV HCoV-NL63	18	2 w–1 y
			β-CoV HCoV-OC43	23	2 w–1 y
D1	Qatar	S1-microarray positive persons with camel contact	NA	19	NA
D2		S1-microarray negative persons with camel contact	NA	18	NA
E	The Netherlands	RT-PCR confirmed MERS case-patients‡	Acute‡	21	1–14 d
F			Convalescent‡	7	15–228 d
G	South Korea	RT-PCR confirmed MERS case-patients	Mild infection§	17	6–12 mo
H			Severe infection¶	15	6–12 mo

*Cohorts A–C were established to test assay specificity; cohorts D–H were established to test assay sensitivity. CoV, coronavirus; CMV, Cytomegalovirus; EBV, Epstein-Barr virus; HCoV, human coronavirus; HMPV, human metapneumovirus; MERS, Middle East respiratory syndrome; mo, month; NA, not applicable; PIV, parainfluenza virus; RSV, respiratory syncytial virus.  †Cross-reactivity. ‡Samples taken from 2 case-patients at different time points. §Samples taken from 6 case-patients at different time points. ¶Samples taken from 5 case-patients at different time points.

### Serologic Assays

We tested all serum samples for MERS-CoV neutralizing antibodies using plaque reduction neutralization assay (PRNT). For S1 reactivity, we used a routine ELISA (rELISA; Euroimmun, https://www.euroimmun.com [*15*]), an in-house ELISA (iELISA), and protein microarray (*8*,*23*). For nucleocapsid reactivity, we used luciferase immunoprecipitation assay (N-LIPS) (*24*). For S2 reactivity, we used ELISA (Appendix).

### Statistical Analyses

We evaluated the specificity and sensitivity and predictive values of the assay platforms using serum samples from patients with PCR-diagnosed MERS-CoV infections, respiratory virus–infected patients, and healthy controls. We compared performance of assay platforms to PCR performance using Fisher exact test and used receiver operating characteristic (ROC) curve to compare performance of different platforms. We performed all statistical analyses using GraphPad Prism version 7 (https://www.graphpad.com).

## Results

### Low Antibody Responses following Mild MERS-CoV Infection

**Figure 1:**
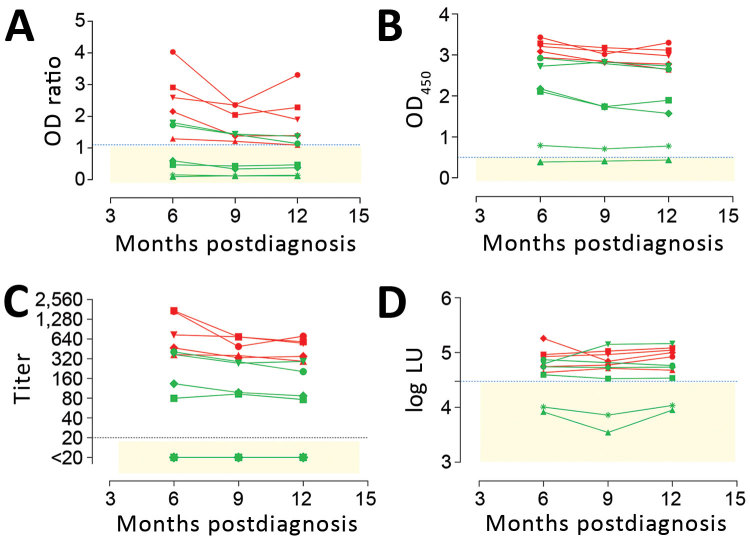
Detection of MERS-CoV–specific antibody responses 6–12 months following PCR-diagnosed mild and severe infections using different assays. Spike S1–specific antibody responses were tested with a routinely used S1 ELISA (rELISA) (A), in-house S1 ELISA (iELISA) (B), and S1 microarray (C). Nucleocapsid-specific antibody responses were tested using a luciferase immunoprecipitation assay (D). Severe infections (red, n = 5; cohort H) resulted in antibody responses detected for up to 1 year by all assays, while detection of mild infections (green, n = 6; cohort G) varied among assays. Horizontal dotted line indicates cutoff for each assay; yellow shaded area indicates serum undetected by each assay. CoV, coronavirus; LU, luminescence units; MERS, Middle East respiratory syndrome; OD, optical density.

Several studies have proposed that antibody levels and longevity following MERS-CoV infection are dependent on disease severity (*5*,*15*,*17*). Among PCR-confirmed MERS patients, mild infections may result in undetectable or lower, short-lived immune responses when compared with severe infections. We evaluated MERS-CoV–specific antibody responses in severe and mild MERS-CoV infections using serum samples collected 6, 9, and 12 months after disease onset from PCR-confirmed MERS-CoV patients from the 2015 South Korea outbreak, 6 with severe and 5 with mild infections (*17*). First, we tested serum samples for MERS-CoV S1 antibodies using different assay platforms (Figure 1; Appendix Table). Consistent with the earlier report (*17*), the routinely used rELISA detected only 2/6 mild infections (Figure 1, panel A). In contrast, iELISA detected 5/6 mild infections (Figure 1, panel B)**.** Similar results were obtained using the S1 protein microarray to screen for MERS-CoV–specific antibodies (Figure 1, panel C). Although these serum samples lacked MERS-CoV neutralizing antibodies (*17*), the presence of nucleocapsid antibodies up to 1 year postinfection in 4/6 mildly infected patients’ samples confirmed the results of the S1 ELISA with an assay targeting another MERS-CoV protein (Figure 1, panel D). All severe cases, on the other hand, were found positive in all tested platforms up to 1 year after disease onset, indicating a robust immune response of high antibody titers in severe cases (Figure 1; Appendix Table). Compared with milder infections, both S1 and neutralizing antibody responses were higher in severely infected cases, confirming that antibody responses are lower following nonsevere infection.

### Specificity and Sensitivity of In-house S1 ELISA and Microarray

To confirm that the variation in the detection of mild cases is caused by the sensitivity of the different platforms used, we further validated the platforms for specificity and sensitivity using 292 serum samples (Table 1). Using MERS-CoV neutralization as the standard for MERS-CoV serology, we tested all serum samples using plaque reduction neutralization assay (PRNT_90_) and for S1, S2, and nucleocapsid reactivities.

**Figure 2:**
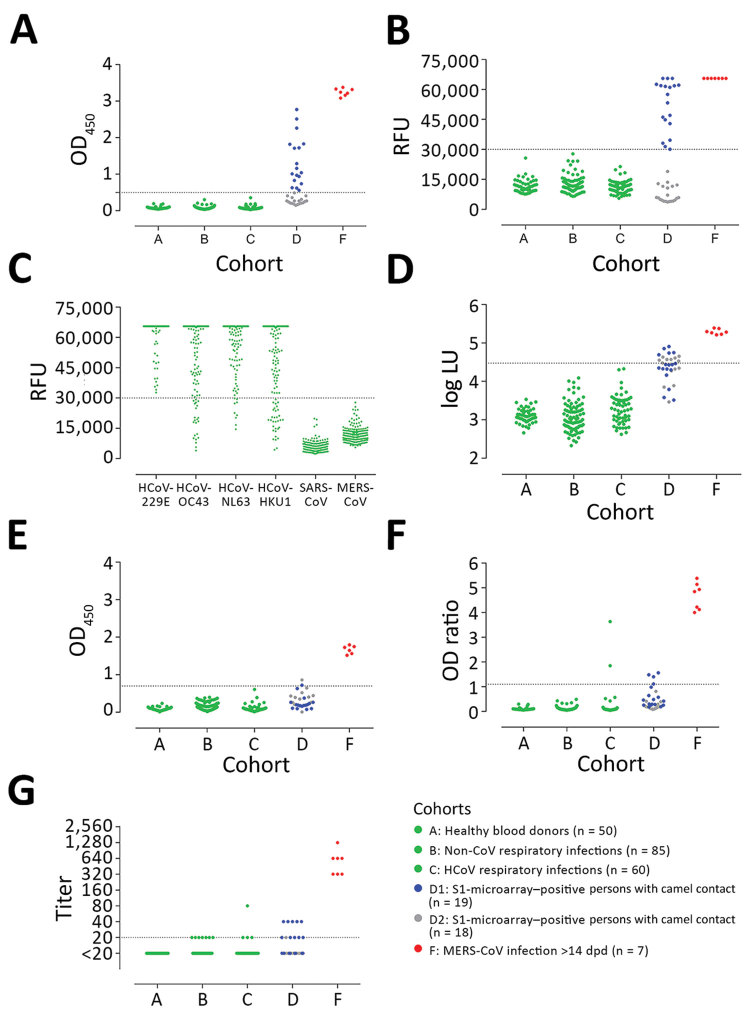
MERS-CoV–specific antibody responses detected by different assay platforms. A) In-house IgG of S1 ELISA (iELISA); B) MERS-CoV S1 protein microarray; C) HCoV S1 microarray reactivity of non-MERS-CoV–infected serum samples to the S1 proteins of 6 different HCoVs; D) nucleocapsid-luciferase immunoprecipitation assay; E) IgG S2 ELISA; F) routinely used IgG S1 ELISA expressed as the ratio of optical density of sample to kit calibrator; G) plaque reduction neutralization test (PRNT), expressed as endpoint titer for 90% plaque reduction. Serum samples tested were obtained from healthy blood donors (n = 50, cohort A); patients with PCR-diagnosed respiratory infections including human coronaviruses (n = 145, cohorts B and C); S1-microarray positive (n = 18, cohort D1) and negative (n = 19, cohort D2) camel contacts; and longitudinal serum samples from 2 PCR-confirmed MERS-CoV–infected patients taken 15–228 days after diagnosis (n = 7, cohort F). Cohort E is not included because patients in this cohort were in the acute phase of infection (<14 days postdiagnosis), in which seroconversion may not have occurred. Cohorts A, B, C, and F are from the Netherlands, cohort D from Qatar. Serum samples were tested at dilutions 1:101 for ELISA and N-LIPS, 1:20 for S1 microarray, and 1:20 to 1:2,560 for PRNT. Dotted lines indicate cutoff for each assay. CoV, coronavirus; LU, luminescence units; MERS, Middle East respiratory syndrome; OD, optical density; RFU, relative fluorescence units.

We assessed the specificity of the assays using serum samples from cohorts A–C: healthy blood donors (cohort A), patients with PCR-confirmed acute respiratory non-CoV infections (cohort B), and patients with acute to convalescent PCR-confirmed α- and β-HCoV infections (cohort C). None of the serum samples from specificity cohorts A–C were reactive by iELISA at the set cutoff, indicating 100% specificity **(**Figure 2, panel A; Appendix). We also evaluated the sensitivity for detecting MERS-CoV infections; iELISA was able to detect MERS-CoV infections among persons with camel contact (cohort D1) who had low antibody levels as determined by protein microarray (*8*). Using samples from acute-phase PCR-diagnosed patients (cohort E), we detected seropositivity 6–8 days postdiagnosis (dpd). All convalescent-phase serum samples (cohort F) were positive up to the last time point tested: 228 dpd for patient 1 and 44 dpd for patient 2 (Appendix Figure 1). 

These results reveal the high specificity and sensitivity of this ELISA platform, supporting our earlier findings and confirming the sensitivity of our platform in detecting low immune responses among cases of milder infection (cohort G) (Figure 1). Overall, iELISA was 100% (95% CI 98.07%–100%) specific and 92.3% (11/13; 95% CI 66.7%–99.6%) sensitive for detection of PCR-confirmed cases (96.9% overall in the tested cohorts; 95% CI 84.3%–99.8%) (Table 2). Moreover, the iELISA performance was in accordance with that of the MERS-CoV S1 protein microarray (Figure 2, panel B). S1 microarray validation showed the same pattern of specificity with no false positives (100% specificity, 95% CI 98.07%–100%) in cohorts A–C and was 84.6% sensitive (95% CI 57.8%–97.3%) for PCR-confirmed cases and 93.8% overall (95% CI 79.9%–98.9%). Specificity of S1 as an antigen for MERS-CoV serology was further supported by the rates of seropositivity of all the serum samples from cohorts A–C: 87.4% for HCoV-HKU1, 91.3% for HCoV-OC43, 96.4% for HCoV-NL63, and 100% for HCoV-229E, as determined by microarray (Figure 2, panel C). All samples were seronegative for SARS-CoV, and no MERS-CoV false positives were detected in the iELISA and microarray. Overall, these results provided evidence for the use of S1 as a specific antigen for MERS-CoV serology.

**Table 2:** Specificity and sensitivity of assay platforms for the detection of MERS-CoV antibodies among PCR-confirmed cases*

Test characteristic	In-house S1 ELISA	S1 microarray	PRNT_90_	Routinely used S1 ELISA
p value	<0.0001	<0.0001	<0.0001	<0.0001
Sensitivity, N = 13				
No. tested positive	12	11	9	9
n/N value (95% CI)	0.9231 (0.6669–0.9961)	0.8462 (0.5777–0.9727)	0.692 (0.4237–0.8732)	0.6923 (0.4237–0.8732)
Specificity, N = 195				
No. tested positive	0	0	1	2
n/N value (95% CI)	1 (0.9807–1)	1 (0.9807–1)	0.9949 (0.9715–0.9997)	0.9897 (0.9634–0.9982)
Positive predictive value				
Value (95% CI)	1 (0.7575–1)	1 (0.7412–1)	0.9 (0.5958 –0.9949)	0.8182 (0.523–0.9677)
Negative predictive value				
Value (95% CI)	0.9949 (0.9717–0.9997)	0.9898 (0.9637–0.9982)	0.9798 (0.9492–0.9921)	0.9797 (0.949–0.9921)
Positive likelihood ratio	NA	NA	135	67.5

Area under the ROC curve				
Area	1	0.9893	0.7348	0.9481
SE	0	0.005409	0.07513	0.01767
95% CI	1–1	0.9787–0.9999	0.5876–0.8821	0.9134–0.9827
p value	<0.0001	<0.0001	<0.0001	<0.0001

*p value calculated by Fisher exact test. CoV, coronavirus; MERS, Middle East respiratory syndrome; NA, not applicable; PRNT, plaque reduction neutralization test; PRNT_90_, 90% endpoint PRNT; ROC, receiver operating characteristic.

We evaluated nucleocapsid and S2 antibody responses after MERS-CoV infections. At the set cutoff, none of the control serum samples tested positive for nucleocapsid antibodies (Figure 2, panel D). We detected seroconversion by nucleocapsid-luciferase immunoprecipitation assay among all severely infected, 4/6 (66.7%) mildly infected, and 5/18 (28%) asymptomatic S1-positive persons with camel contact. When testing for MERS-CoV S2–specific antibody responses, none of the control serum samples in cohorts A–C was cross-reactive (Figure 2, panel E), whereas 1/17 S1-negative samples and 1/18 S1-positive samples from persons with camel contact tested positive. These findings indicate low immune responsiveness in mild MERS cases. Thus, when comparing the use of S1, S2, and N proteins for the detection of MERS-CoV infections, S1 showed the highest specificity and sensitivity among the 3 tested proteins.

### rELISA Validation 

**Figure 3:**
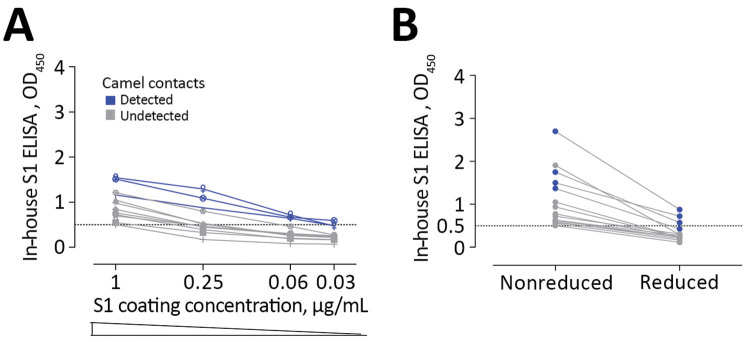
Low sensitivity of commercial S1 ELISA shown as the effect of lowering coating antigen concentration (A) or antigen denaturation (B) on the sensitivity of antibody detection among Middle East respiratory syndrome coronavirus–infected persons with camel contact. All samples were seropositive by in-house S1 ELISA and microarray. Dark blue indicates those that tested seropositive by commercial S1 ELISA.

Strikingly, the routinely used ELISA showed the least sensitivity among the tested S1 platforms (Table 2; Figure 1; Figure 2, panel F). We saw this difference in the cohort of persons with camel contact from Qatar who had mild to asymptomatic infections and who were identified to be seropositive for MERS-CoV in an earlier study (*8*) (Figure 2, panel F, cohort D1). Although they tested seropositive by iELISA and the microarray platform, only 20% of those also tested positive using the rELISA platform. We tested different coating conditions and found that a reduction in antigen coating or a loss of some conformational epitopes could have contributed to the low sensitivity seen in the rELISA versus the iELISA, despite testing the same antigen (S1) (Figure 3). This low sensitivity confirms the likelihood of false-negative detection of some MERS-CoV cases using rELISA.

**Figure 4:**
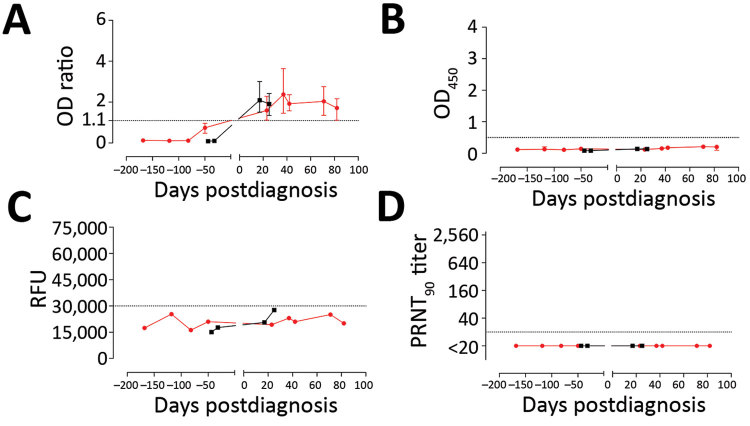
Reactivity to Middle East respiratory syndrome coronavirus of serum samples from 2 patients with human coronavirus OC43 in different assays. Longitudinal serum samples, collected before and after OC43 infection, from the 2 patients (red, patient 1; black, patient 2) were analyzed by commercial IgG S1 ELISA (A); in-house IgG S1 ELISA (B); S1 protein microarray(C); and PRNT_90_ (D). Dotted line indicates the cutoff for each assay. Error bars indicate 95% CIs. OD, optical density; PRNT_90_, 90% reduction in plaque reduction neutralization test; RFU, relative fluorescence units.

We evaluated the specificity of the rELISA platform using cohorts A–C. Among these, serum samples from 2 patients with HCoV-OC43 (a β-CoV) infection tested positive (Figure 2, panel F) but tested negative for MERS-CoV neutralization by PRNT_90_ and S1 antibodies by iELISA and microarray (Table 3). Thus, to confirm the cross-reactivity of the 2 serum samples with MERS-CoV S1 in rELISA, we tested serum samples taken from both patients at different time points, before and after OC43 infection. All preinfection serum samples were negative, but all postinfection serum samples were positive in the rELISA (Figure 4). On the contrary, none of the serum samples was positive when tested by PRNT, Western blot, immunofluorescence assay, iELISA, or S1 protein microarray (using commercial and in-house S1 proteins), indicating a false-positive reaction in the rELISA. Overall, the rELISA was 98.97% (95% CI 96.3%–99.8%) specific in the tested cohorts **(**Table 3). Using a lower cutoff (optical density ratio 0.4) to show 100% specificity and sensitivity as suggested in an earlier study (*30*), did increase the sensitivity, (from 69.2% to 84.6%), but doing so reduced specificity; numbers of false-positive results increased from 2 to 7 and specificity decreased from 98.97% to 96.4% (Appendix Figure 2).

**Table 3:** Sensitivity and specificity results of routinely used commercial S1 ELISA and PRNT_90_ assays for MERS-CoV*

Assay parameter and sample source	Infection	No. positive/no. tested					
		S1 rELISA†	PRNT_90_ (titer)			Specificity or sensitivity, %	
			S1-positive	S1-negative		S1 rELISA	PRNT_90_
Specificity						98.97	93.33 (1:20); 99.5 (1:40)
Healthy blood donors	None	0/50	NA	0/50			
Non-CoV respiratory infections	Adenovirus	0/5	NA	1/5(20)			
	Bocavirus	0/2	NA	0/2			
	Enterovirus	0/2	NA	0/2			
	HMPV	0/9	NA	1/9 (20)			
	Influenza A	0/13	NA	4/13 (20, 20, 20, 20)			
	Influenza B	0/6	NA	0/6			
	Rhinovirus	0/9	NA	2/9 (20, 20)			
	RSV	0/9	NA	1/9 (20)			
	PIV-1	0/4	NA	0/4			
	PIV-3	0/4	NA	0/4			
	*Mycoplasma*	0/1	NA	0/1			
	CMV	0/9	NA	0/9			
	EBV	0/12	NA	0/12			
Recent CoV infections‡	α-CoV HCoV-229E	0/19	NA	3/19 (20, 20, 20)			
	α-CoV HCoV-NL63	0/18	NA	0/18			
	β-CoV HCoV-OC43	2/23	0/2	1/21 (80)			
Sensitivity							
Persons with camel contact	S1-microarray positive§	4/19	4/4 (40, 40, 40, 20)	6/15 (40, 40, 20, 20, 20, 20)		21	52.6
	S1-microarray negative	0/18	NA	1/18 (20)		NA	NA
RT-PCR–confirmed MERS cases	<14 d postdiagnosis	11/21	11/11	1/10 (80)		NA	NA
	>14 d postdiagnosis	7/7	7/7	NA		100	100
	6–12 mo postdiagnosis; mild infection	5/17	5/5	NA		35.3	35.3
	6–12 mo postdiagnosis; severe infection	15/15	15/15	NA		100	100

*CMV, cytomegalovirus; CoV, coronavirus; EBV, Epstein-Barr virus; ELISA, enzyme-linked immunosorbent assay; HCoV, human coronavirus; HMPV, human metapneumovirus; MERS, Middle East respiratory syndrome; NA, not applicable; PIV, parainfluenza virus; PRNT, plaque reduction neutralization test; PRNT_90_, 90% endpoint PRNT; rELISA, routine ELISA; RSV, respiratory syncytial virus; RT-PCR, reverse transcription PCR.  †None of the serum samples from specificity cohorts tested positive by in-house S1 ELISA or microarray. ‡Cross-reactivity. §All 19 serum samples (protein microarray positive) tested positive by in-house S1 ELISA.

### Mild MERS-CoV infections and Neutralizing Antibodies

To investigate the difference in the neutralization responses produced following severe and mild infections and the reliability of neutralization assays as confirmatory assays for mild infections, we validated PRNT_90_ for specific and sensitive detection of MERS-CoV infections. Although none of the healthy blood donors (cohort A) were reactive, the respiratory patients (cohorts B and C) showed low levels of cross-neutralization (titer 20) in 12 serum samples. One sample with a titer of 80 (Figure 2, panel G) was from an HCoV-OC43 patient; none of the serum samples taken at 4 earlier time points from that patient showed any neutralization by PRNT (data not shown). All 13 serum samples tested negative for S1 antibodies in all tested platforms (Table 3); none of the serum samples was positive in 2 assays. For PCR-diagnosed MERS cases (cohorts E–H), PRNT_90_ showed 100% sensitivity for detecting severe cases after the seroconversion period (>14 dpd; cohort F) and for up to 1 year (cohort H), indicating strong neutralizing antibody responses. 

In contrast, results varied for mild cases (cohort G). Neutralizing antibodies were detected in 3/6 (50%) of mild infections (Appendix Table 1), highlighting lower, shorter-lived neutralizing responses among mild cases. This finding is consistent with the results of a cohort of mild to asymptomatic MERS-CoV–infected persons with camel contact from Qatar (*8*) (Figure 2, panel G, cohort D1). These persons had low to undetectable neutralizing antibodies while being reactive to S1 on the protein microarray platform and in our iELISA. 

### Nonneutralizing Antibodies after Mild MERS-CoV Infections

**Figure 5:**
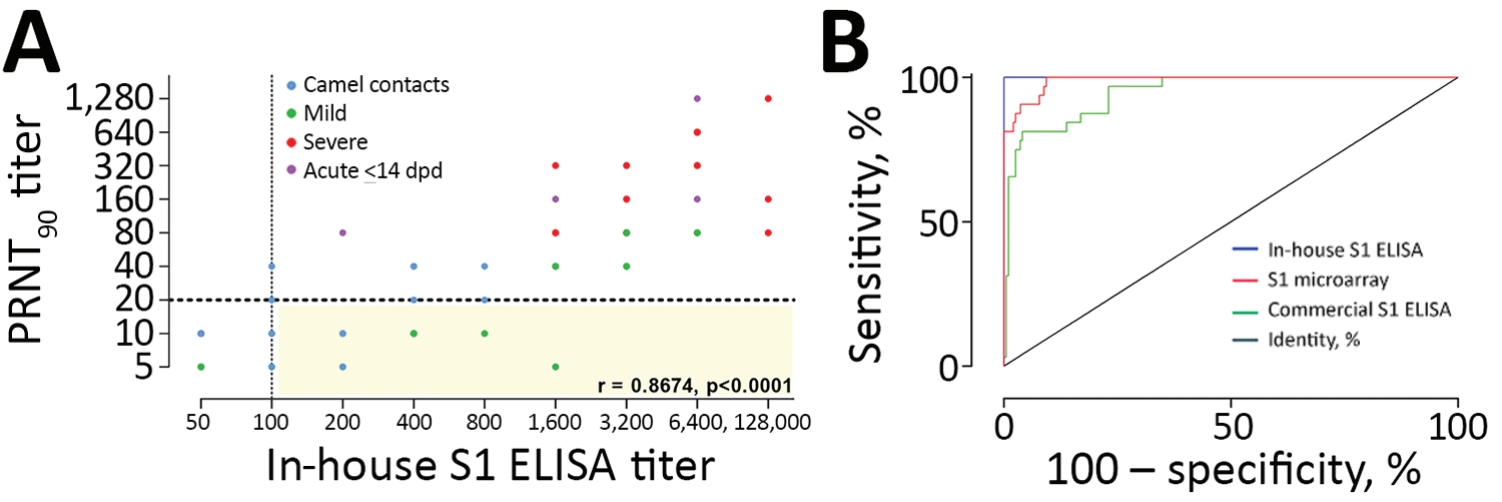
Correlation between neutralizing and S1 antibody responses and comparison of different S1 platforms. A) PRNT_90_ neutralization titers and IgG titers obtained by in-house S1 ELISA among PCR-confirmed MERS-CoV patients and persons with camel contact. Spearman correlation r value and 2-tailed p-value are shown. Yellow shading indicates S1-reactive nonneutralizing antibodies. B) Receiver operator characteristic (ROC) curves comparing the specificity and sensitivity of different MERS-CoV S1–based platforms for the diagnosis of MERS-CoV infections among PCR-confirmed cases. AUC for iELISA (blue) is 1; for S1 microarray (red) is 0.9893; for rELISA (green) is 0.9481. Dotted lines show the cutoff for each assay. AUC, area under the curve; dpd, days postdiagnosis; PRNT_90_, 90% reduction in plaque reduction neutralization test.

For the PCR-confirmed MERS-CoV patients (cohorts E–H) and serologically positive persons with camel contact (cohort D1), S1 antibody titers as determined by iELISA strongly correlated with neutralization titers **(**Figure 5, panel A), showing that S1 antibody response is a reliable predictor of neutralization activity. This finding indicates that a population of mildly infected patients with S1-reactive antibodies but no detectable neutralizing antibodies could easily be missed in attempts to confirm cases by neutralization assay.

## Discussion

Serologic detection of MERS-CoV exposure is valuable for identifying asymptomatic cases and virus reservoirs in population screening and epidemiologic studies, as well as for contact investigations. Detection aids in understanding the host immune response to the virus, identifying key viral immunogens, and mapping key neutralizing antibodies, which all lead to implementing appropriate preventive and therapeutic measures. Antibody responses varied among PCR-confirmed MERS-CoV cases; case-patients with mild and asymptomatic infections showed low or undetectable seroconversion, in contrast to severe infections that resulted in robust responses (*5*,*17*,*31*). The low-level antibody responses produced following nonsevere infections led to failure in detecting such responses in some patients by a routinely used ELISA and neutralization assays (*5*,*17*,*32*). This result may have impeded estimation of prevalence of virus infections in surveillance studies. We were able to detect nonneutralizing antibody responses among previously infected mild and asymptomatic cases that were previously unidentified; this finding indicates that MERS-CoV prevalence could be higher than current estimates and that using sensitive platforms could lead to more precise calculation of incidence rates.

Although an earlier study evaluating serologic responses among PCR-confirmed MERS patients reported seroconversion in only 2/6 (33%) mildly infected cases (*17*), we were able to detect 5/6 (83.5%) by our in-house S1 ELISA and 4/6 (67%) by microarray. S1 iELISA and microarray were highly sensitive for detecting MERS-CoV infections, showing 100% specificity in the tested cohorts. Although the rELISA platform detected severe infections with no false negatives, it did not detect seroconversion among some mildly infected PCR-confirmed and asymptomatic persons with camel contact who had low antibody responses. In addition, rELISA results showed cross-reactivity with some serum samples from HCoV-OC43–infected persons. The variation in the reactivity between the 2 ELISA platforms could be attributed to the difference in the coating protein preparations used in each or to the reduced stability of the protein during storage of the rELISA platform. 

Overall, our results validate the use of S1 as a specific antigen for MERS-CoV serology if folding is correct, providing a highly specific 1-step diagnostic approach without false positives omitting the need for a confirmatory assay. In particular, neutralizing antibodies were undetectable after most asymptomatic and some mild infections. Using 50% instead of 90% reduction as a cutoff for PRNT can increase the sensitivity of the assay for confirming mild or asymptomatic infections (*15*,*21*,*33*), but it is crucial to precede PRNT with a sensitive screening assay to avoid false-negative results.

Prolonged viral shedding observed in severely infected patients but not in patients with mild infections (*5*,*17*,*34*) indicated that a short-lived infection in nonsevere cases may account for lower antibody responses, including functional neutralizing antibodies. A possible reason is that nonneutralizing antibodies comprise a substantial proportion of antibodies elicited after a viral infection; these antibodies can be elicited against viral proteins, including immature forms of surface proteins, released through lysis of infected cells following a short-lived abortive infection (*35*,*36*). We found that spike antibody titers were produced at higher titers than nucleocapsid antibodies and neutralizing antibodies were undetectable following nonsevere infections. These findings indicate that anti-spike antibodies are more sensitive predictors for previous MERS-CoV infections, especially mild and asymptomatic infections, and that conducting neutralization assays to confirm serologic findings, as recommended by the World Health Organization (*37*), could result in potential underestimation of the true prevalence in epidemiologic studies. 

Further studies testing patients with previously indeterminate infection could provide further clues on the epidemiology of MERS-CoV. A recent study reported the presence of MERS-CoV–specific CD8+ T-cell responses after MERS-CoV infection, irrespective of disease severity (*38*). Therefore, T-cell assays can be used to confirm serologic findings in epidemiologic studies (mainly asymptomatic cases) instead of neutralization assays that could yield underestimated results. However, further studies are needed to rule out possible T-cell cross-reactivity with other HCoV.

Despite the use of 90% reduction as endpoint for PRNT, we observed cross-neutralization in the respiratory panel samples (13/195). All but 1 sample had a titer of 20, and all 13 were S1-negative. We reported a similar finding in an earlier study, where 1 of 35 S1 negative serum samples had a neutralization titer of 20 (*8*). This finding was unexpected because neutralization assays, with their high specificity, are considered the standard for MERS-CoV serodiagnosis. Such seemingly false positives could be attributed to the presence of natural antibodies or cross-reactive HCoV antibodies (*15*,*32*,*35*,*39*). 

Cross-neutralization among human coronaviruses has rarely been reported. Chan et al. described cross-neutralization between SARS-CoV and MERS-CoV at low titers (<20) (*32*). However, these serum samples also tested positive for HCoV-OC43 neutralization. This finding, along with ours, raises the probability that HCoV-OC43 antibodies caused cross-reactivity; antibodies in the serum sample could be recognizing an epitope outside S1 and thus not detected in ELISA. Of interest, we detected an HCoV-OC43 patient serum sample that could neutralize MERS-CoV at PRNT_90_ titer <80, but we found that the patient received an oncolytic medication shown to have antiviral activity (*40*). This finding could also be a probable reason for the observed cross-neutralization. Overall, while serum samples from healthy blood donors showed no cross-neutralization or cross-reactivity to S2 or N proteins, we observed some cross-neutralization and comparably higher reactivity to S2 and N proteins in serum samples of patients with respiratory infections, which we did not detect by our in-house S1 platforms. Thus, we could not avoid cross-reactivity to S2 and N proteins, leading to false positives, without loss of sensitivity. The high specificity of the S1 protein enabled us to set a cutoff high enough to ensure specificity without losing sensitivity.

Using S1 in optimized platforms enabled us to detect seroconversion among otherwise unrecognized nonsevere MERS-CoV cases with very high sensitivity and 100% specificity. Our findings indicate that our iELISA and microarray for MERS-CoV diagnostics (Table 2; Figure 5, panel B) could be reliable diagnostic tools for identifying MERS-CoV infections. For further standardization of the assay, a calibrator (e.g., monoclonal antibody) can be included in each run to avoid intraassay variations. 

Although further testing on a larger cohort may be required to rule out cross-reactivity, ensure sensitivity, and thereby validate general use as a 1-step diagnostic assay, the data obtained in this study indicate that cross-reactivity between HCoVs (at least when testing for MERS-CoV and SARS-CoV reactivity) is unlikely to occur when using optimized platforms with the divergent S1 protein. A more recent follow-up study revealed that, among 454 serum samples tested using our in-house S1 ELISA, including those from persons with camel contact, only 2 samples, both MERS-CoV–neutralization positive, tested positive whereas all other serum samples were found to be negative in the iELISA (R. Bassal et al., unpub. data). Thus, in principle, low-level antibody responses among nonsevere MERS-CoV cases may be revealed by a single ELISA test. 

Because patients with mild or asymptomatic infections do not develop severe illness and thus go unrecognized, they might play a role in spreading the virus into the community, initiating outbreaks in which index case-patients report no history of camel or patient exposure. Therefore, defining the subclinical burden of infection will enable better understanding of the extent, severity, and public health threat posed by MERS-CoV, which, in turn, will guide the development and implementation of proper strategies to contain and prevent ongoing outbreaks of infection with this virus.

Appendix Additional information about sensitive and specific detection of low-level antibody responses in mild MERS-CoV infections.
