# Supplementary material for: Sensitive and Specific Detection of Low-Level Antibody Responses in Mild Middle East Respiratory Syndrome Coronavirus Infections
Source: Emerg Infect Dis. 2019 Oct;25(10):1868–77. doi: 10.3201/eid2510.190051 (PMC6759241; doi:10.3201/eid2510.190051)
Supplement: Appendix — Additional information about sensitive and specific detection of low-level antibody responses in mild MERS-CoV infections. [file 19-0051-Techapp-s1.pdf]

Article DOI: https://doi.org/10.3201/eid2510.190051

Sensitive and Specific Detection of Low-Level Antibody Responses in Mild Middle East Respiratory Syndrome Coronavirus Infections

Appendix

Serum Samples

We collected serum samples from 50 healthy blood donors (Cohort A) as negative controls Sanquin Blood Bank (Rotterdam, the Netherlands) obtained written informed consent for research use. We assessed specificity using cohorts B and C, which consisted of 145 serum specimens from patients confirmed by RT-PCR to be positive for human respiratory infections. Those included samples from persons recently infected with 13 different respiratory viruses, or with acute IgM-positive CMV and EBV infection, which are known to be cross-reactive in serologic assays; as well as *Mycoplasma pneumoniae*. Because MERS has no pathognomonic signs distinguishing it from other respiratory infections, cohort B, including 85 samples from acute non-HCoV respiratory infections, was used to assess specificity in acute cases. To further evaluate specificity, cohort C included serum samples from acute to convalescent HCoV-NL63, –229E, and -OC43 patients. Furthermore, we assessed Cohorts D-G were used to evaluate sensitivity of the different platforms. Serologically identified MERS-CoV–infected mild and asymptomatic persons with camel contact (D1) and healthy donors (D2) from Qatar characterized in an earlier study (*1*) constituted cohort D. Serial serum samples from 2 RT-PCR–diagnosed MERS-CoV case-patients imported to the Netherlands (*2*) were used as positive controls and to evaluate antibody kinetics; these were 15 serum samples from patient 1, ranging 4–228 days postdiagnosis (dpd), and 13 from patient 2, ranging 1–44 dpd. These 28 samples were categorized into cohorts E (<14 dpd, acute phase) and F (>14 dpd, convalescent phase). Finally, samples from the PCR-diagnosed mild (cohort G) and severely (cohort H) infected MERS-CoV case-patients from South Korea, described earlier (*34*), formed the last 2 cohorts.

All samples were stored at −20°C until use. The use of serum samples from the Netherlands was approved by the local medical ethical committee (MEC approval: 2014–414). The Institutional Ethics Review Board of Seoul National University Hospital approved use of samples from South Korea (approval no. 1506–093–681). The Ethics and Institutional Animal Care and Use Committees of the Medical Research Center, Hamad Medical Corporation, approved the use of samples from Qatar (permit 2014–01–001).

Protein Expression

We expressed MERS S1 (amino acids 1–751) in HEK-293T cells as a C-terminal human IgG Fc (hFc) tagged protein. We purified S1-hFc protein using protein A sepharose beads (ThermoFisher Scientific, https://www.thermofisher.com) and cleaved off the hFc domain using Factor Xa (EMD Millipore, http://www.emdmillipore.com). We used X-arrest Agarose (EMD Millipore, http://www.emdmillipore.com) to obtain soluble S1 after removal of Factor Xa; S1 protein was used for coating our in-house S1 ELISA plates and microarray.

Spike S1 proteins of other HCoVs, –HKU1 (residues 1–750), –OC43 (residues 1–760), NL63 (residues 1–717), 229E (residues 1–537), and SARS-CoV (residues 1–676), were expressed as C-terminal murine IgG2a Fc tagged proteins as described earlier (*5*) and used for coating S1 protein microarray.

Recombinant MERS-CoV spike S2 subunit (amino acids 752–1262) was produced in the baculovirus expression system. Briefly, we cloned the codon-optimized MERS-CoV S2 encoding sequence into the pFastbac transfer vector (Invitrogen, https://www.thermofisher.com) in-frame between honeybee melittin (HBM) secretion signal peptide and a triple StrepTag purification tag. We produced acmid DNA and recombinant baculovirus according to protocols from the Bac-to-Bac system (Invitrogen). We expressed MERS-CoV S2 protein in Sf-9 cells after infection with the recombinant baculovirus. We harvested recombinant S2 from cell culture supernatants 3 days postinfection and purified it using StrepTactin sepharose affinity chromatography (IBA, https://www.iba-lifesciences.com). The protein was used to coat ELISA plates.

PRNT

PRNT was used as a reference for this study, because it is the standard for MERS-CoV serology. We tested serum samples for their neutralization capacity against MERS-CoV (Erasmus MC isolate) by plaque-reduction neutralization test (PRNT) using Huh-7 cells in a 96-well plate format. Heat-inactivated samples were 2-fold serially diluted (1:20 up to 1:2560) in RPMI1640 medium supplemented with penicillin, streptomycin, and 1% fetal bovine serum, starting at a dilution of 1:10 in 50 μL. We added 50 μL of the virus suspension to each well (500 $TCID_{50}$) and incubated at 37°C for 1 h. Following incubation, we transferred the mixtures (virus and serum) on Huh-7 cells cultured in 96-well plates and incubated them at 37°C for 8 h. We then fixed the cells and stained them with immunofluorescent staining. The serum neutralization titer is the reciprocal of the highest dilution resulting in an infection reduction of $\geq$90% ($PRNT_{90}$). A titer of $\geq$20 was considered to be positive.

S1 ELISA

We performed MERS-CoV IgG S1 ELISA using a commercial kit (Euroimmun, https://www.euroimmun.com) and performed the assay according to manufacturer's protocol. The optical density (OD) was measured at 450 nm, and a ratio of the reading of each sample to the reading of the calibrator, included in the kit, was calculated for each sample (OD ratio). According to the manufacturer's guidelines, samples with an OD ratio <0.8 were considered negative, those with an OD ratio >1.1 were considered positive, whereas those in between were considered borderline.

We performed inhouse S1 ELISA by coating 96-well microtiter ELISA plates with MERS-CoV S1 protein (1 μg/mL) in PBS overnight at 4°C. Following blocking, diluted serum (1:100 or 2-fold serially diluted [1:100–1:12800] for titers) were added and incubated at 37°C for 1h. Bound antibodies were detected using peroxidase-labeled rabbit anti–human IgG (Dako, https://www.agilent.com) and TMB as a substrate. The absorbance of each sample was measured at 450 nm. We set a cutoff at 0.5, which is >6 standard deviations above the mean value of the negative respiratory cohort (0.46). Serum titers correspond to the reciprocal of the highest dilution giving a signal above the cutoff. We tested all samples twice in 2 independent assays.

S1 Protein Microarray

We diluted serum 4-fold (1:20 to 1:1280) and tested using an HCoV S1 protein microarray as previously described (*6*); including S1 domains of the 6 known HCoVs. We set a cutoff was set at 30,000 relative fluorescent units and determined serum titers as the reciprocal of the serum dilution corresponding to the EC_{50} of each serum sample interpolated from a concentration-response curve (*7*).

N-LIPS Assay

Anti-nucleocapsid antibody responses were tested using a luciferase immunoprecipitation assay (LIPS). The N protein was expressed in HEK-293T cells as an N terminal *Renilla* luciferase (Ruc)-tagged protein (Ruc-N) using pREN2 expression vector kindly provided by Dr. Peter D. Burbelo (*8*). The cells were harvested in lysis buffer (50 mM Tris [pH 7.5], 100 mM NaCl, 5 mM MgCl2, 1% Triton X-100, 50% glycerol, protease inhibitors), and the luminescence units (LU) per µL was used as a measure of antigen concentration in cell lysates. We conducted LIPS assay according to Burbelo et al. (*9*) with minor modifications. Briefly, we diluted serum 1:10 in buffer A (50 mM Tris [pH 7.5], 100 mM NaCl, 5 mM MgCl2, 1% Triton X-100). Then we incubated a mixture containing 10 µL of diluted serum and 1× 107 RLU of Ruc-Ag in a total volume of 100 µL of buffer A per well at room temperature on a rotary shaker for 1 h. We transferred the mixture containing antigen-antibody complex (100 µL) into MultiScreenHTS BV Filter Plate (Merck Millipore, https://www.merckmillipore.com) containing 5 µL of a 30% suspension of UltraLink protein A/G beads and reincubated under the same conditions for 1 more hour. After that, the wells were washed 8 times with buffer A and twice with PBS and luminescence was measured for each well after adding 100µL of 0.1 µM coelenterazine (Nanolight Technology, www.nanolight.com) in assay buffer (50 mM potassium phosphate, pH 7.4, 500 mM NaCl, 1 mM EDTA). We tested serum samples in duplicates in $\geq$2 independent assays and averaged the data to determine the LU value for each sample. A cutoff was set at 30,000 LU.

S2 ELISA

We conducted MERS-CoV IgG S2 ELISA following the same protocol used for the inhouse S1 ELISA. A cutoff was set at 0.72, which is equal to 6 standard deviations above the mean value of the negative cohorts.

References

1. Reusken CB, Farag EA, Haagmans BL, Mohran KA, Godeke GJ V, Raj S, et al. Occupational exposure to dromedaries and risk for MERS-CoV infection, Qatar, 2013–2014. Emerg Infect Dis. 2015;21:1422–5. PubMed https://doi.org/10.3201/eid2108.150481

2. Fanoy EB, van der Sande MA, Kraaij-Dirkzwager M, Dirksen K, Jonges M, van der Hoek W, et al. Travel-related MERS-CoV cases: an assessment of exposures and risk factors in a group of Dutch travellers returning from the Kingdom of Saudi Arabia, May 2014. Emerg Themes Epidemiol. 2014;11:16. PubMed https://doi.org/10.1186/1742-7622-11-16

3. Choe PG, Perera RAPM, Park WB, Song KH, Bang JH, Kim ES, et al. MERS-CoV antibody responses 1 year after symptom onset, South Korea, 2015. Emerg Infect Dis. 2017;23:1079–84. PubMed https://doi.org/10.3201/eid2307.170310

4. Oh MD, Park WB, Choe PG, Choi SJ, Kim JI, Chae J, et al. Viral load kinetics of MERS coronavirus infection. N Engl J Med. 2016;375:1303–5. PubMed https://doi.org/10.1056/NEJMc1511695

5. Li W, Hulswit RJG, Widjaja I, Raj VS, McBride R, Peng W, et al. Identification of sialic acid-binding function for the Middle East respiratory syndrome coronavirus spike glycoprotein. Proc Natl Acad Sci U S A. 2017;114:E8508–17. PubMed https://doi.org/10.1073/pnas.1712592114

6. Reusken C, Mou H, Godeke GJ, van der Hoek L, Meyer B, Müller MA, et al. Specific serology for emerging human coronaviruses by protein microarray. Euro Surveill. 2013;18:20441. PubMed https://doi.org/10.2807/1560-7917.ES2013.18.14.20441

7. Koopmans M, de Bruin E, Godeke GJ, Friesema I, van Gageldonk R, Schipper M, et al. Profiling of humoral immune responses to influenza viruses by using protein microarray. Clin Microbiol Infect. 2012;18:797–807. PubMed https://doi.org/10.1111/j.1469-0691.2011.03701.x

8. Burbelo PD, Goldman R, Mattson TL. A simplified immunoprecipitation method for quantitatively measuring antibody responses in clinical sera samples by using mammalian-produced Renilla luciferase-antigen fusion proteins. BMC Biotechnol. 2005;5:22. PubMed https://doi.org/10.1186/1472-6750-5-22

9. Burbelo PD, Ching KH, Klimavicz CM, Iadarola MJ. Antibody profiling by luciferase immunoprecipitation systems (LIPS). J Vis Exp. 2009;32:1549. https://doi.org/10.3791/1549

10. Ko JH, Muller MA, Seok H, Park GE, Lee JY, Cho SY, et al. Suggested new breakpoints of anti-MERS-CoV antibody ELISA titers: performance analysis of serologic tests. Eur J Clin Microbiol Infect Dis. 2017;36:2179–86.

11. Drosten C, Meyer B, Müller MA, Corman VM, Al-Masri M, Hossain R, et al. Transmission of MERS-coronavirus in household contacts. N Engl J Med. 2014;371:828–35. PubMed https://doi.org/10.1056/NEJMoa1405858

12. Müller MA, Meyer B, Corman VM, Al-Masri M, Turkestani A, Ritz D, et al. Presence of Middle East respiratory syndrome coronavirus antibodies in Saudi Arabia: a nationwide, cross-sectional, serological study. Lancet Infect Dis. 2015;15:559–64. PubMed https://doi.org/10.1016/S1473-3099(15)70090-3

Appendix Table. Serologic responses of Middle East respiratory syndrome coronavirus, South Korea, 2015

		Euroimmun S1 ELISA OD ratio			In-ouse S1 ELISA OD (titer)			S1 microarray titer			PRNT$_{90}$ titer			N-LIPS LU x 103		
Disease severity	Pt	6 mpi	9 mpi	12 mpi	6 mpi	9 mpi	12 mpi	6 mpi	9 mpi	12 mpi	6 mpi	9 mpi	12 mpi	6 mpi	9 mpi	12 mpi
Severe	C	3.30	2.35	4.03	3.24 (6400)	3.11 (3200)	3.44 (12800)	1685	490	707	640	ND	ND	55.4	59.3	84.2
	D	2.91	2.04	2.28	3.27 (12800)	3.18 (6400)	3.12 (3200)	1734	689	581	ND	ND	80	92.2	106.2	121.3
	F	1.29	1.21	1.09	2.99 (1600)	2.77 (1600)	2.65 (1600)	370	358	293	ND	ND	160	43.3	51.5	47.7
	G	2.59	2.35	1.90	3.21 (3200)	3.19 (3200)	3.01 (3200)	740	693	551	ND	ND	80	84.3	92.3	111.0
	H	2.15	1.38	1.39	3.09 (3200)	2.82 (1600)	2.76 (1600)	468	332	347	ND	ND	40	182.1	69.3	100.6
Mild	K	1.72	NA	1.14	3.06 (3200)	NA	2.64 (1600)	407	NA	203	ND	ND	40	74.2	NA	58.0
	L	0.47	0.43	0.47	2.1 (1600)	1.7 (800)	1.87 (800)	80	93	76	ND	ND	<20	39.4	33.5	27.8
	M	0.10	0.12	0.12	0.4 (<100)	0.41 (<100)	0.45 (<100)	<20	<20	<20	ND	ND	<20	8.3	3.5	9.0
	N	1.80	1.44	1.37	2.67 (6400)	2.84 (3200)	2.74 (3200)	371	274	292	ND	ND	80	61.9	142.4	147.0
	O	0.60	0.34	0.38	2.33 (800)	1.79 (400)	1.58 (400)	133	98	87	ND	ND	20	55.2	53.3	54.5
	P	0.15	0.12	0.14	0.78 (200)	0.67 (200)	0.73 (400)	<20	<20	<20	ND	ND	<20	10.2	7.3	10.8
Severe		5/5	5/5	5/5	5/5	5/5	5/5	5/5	5/5	5/5	5/5	ND	ND	5/5	5/5	5/5
Mild		2/6	2/6	2/6	2/6	5/6	4/5	5/6	4/6	3/5	4/6	ND	ND	3/6	4/6	3/5

*Red type indicates serum samples that tested negative in each assay. LU, luminescence units; mpi, months post-infection; N-LIPS, nucleocapsid luciferase immunoprecipitation assay; OD, optical density; PRNT$_{90}$, >90% plaque-reduction neutralization test; Pt., patient; S1, spike S1 protein.

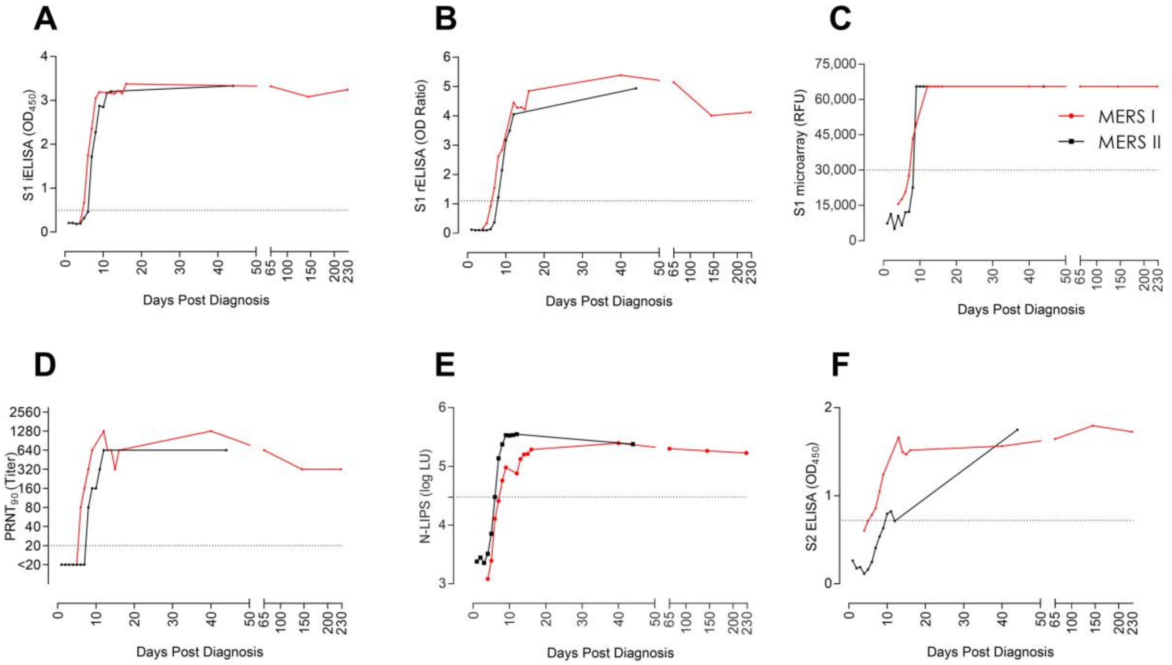

Appendix Figure 1. Kinetics of MERS-CoV specific S1, S2, N and neutralizing antibody responses in 2 patients with Middle East respiratory syndrome. We tested antibody responses in serum samples using (A) an inhouse S1 ELISA (iELISA); (B) a routinely used S1 ELISA (rELISA); (C) S1 microarray; (D) plaque reduction neutralization test (PRNT); (E) nucleocapsid luciferase immunoprecipitation assay (N-LIPS); (F) S2 ELISA. Shown are antibody responses in time for serum from 2 patients (red, patient 1; black, patient 2). The dotted lines show the cutoff for each assay. LU, luminescence units, N-LIPS, nucleocapsid luciferase immunoprecipitation assay; OD, optical density at 450 nm; $PRNT_{90}$, 90% reduction in plaque reduction neutralization test; and RFU, relative fluorescence units.

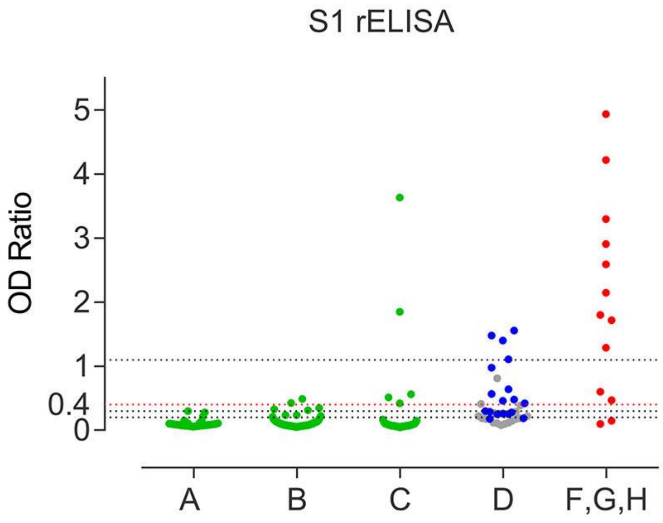

Cut-off (OD ratio)		Specificity		Sensitivity	
**Reference**	Value	**n**	**%**	**n**	**%**
Assay kit	**1.1**	**193/195**	**98.97**	**9/13**	**69.23**
**(10)**	0.4	188/195	96.41	11/13	84.62
**(11,12)**	**0.3**	**185/195**	**94.87**	**11/13**	**84.62**
**(10)**	**0.2**	**177/195**	**90.77**	**11/13**	**84.62**

Appendix Figure 2. Effect of lowering the assay cutoff on the specificity and sensitivity of the rELISA for detection of MERS-CoV specific antibodies. The overall specificity and sensitivity of rELISA in the specificity cohorts A–C and sensitivity cohorts D–H. Graph shows the ratio of optical density of sample to kit calibrator for study cohorts. Dotted lines indicate different cutoffs (OD ratio 1.1, 0.4, 0.3, 0.2). The table shows the number and percentage specificity (cohorts A–C; n = 195) and sensitivity in PCR-confirmed cases (cohorts F, G, H; n = 13). Cohorts: A, healthy blood donors (n = 50); B, acute non-CoV respiratory infections (n = 85); C, acute to convalescent non-MERS-CoV respiratory infections (n = 60); D, S1-microarray positive persons with camel contact (n = 18; blue; D1) and S1-microarray negative persons with camel contacts (n = 19; gray; D2); F–H, PCR-confirmed MERS-CoV–infected patients (n = 13).
